# Transcriptional bursting: from fundamentals to novel insights

**DOI:** 10.1042/BST20231286

**Published:** 2024-08-09

**Authors:** Daniel Hebenstreit, Pradip Karmakar

**Affiliations:** School of Life Sciences, University of Warwick, CV4 7AL Coventry, U.K.

**Keywords:** bursting, enhancers, LLPS, parameter inference, RNA polymerase, transcription

## Abstract

Transcription occurs as irregular bursts in a very wide range of systems, including numerous different species and many genes within these. In this review, we examine the underlying theories, discuss how these relate to experimental measurements, and explore some of the discrepancies that have emerged among various studies. Finally, we consider more recent works that integrate novel concepts, such as the involvement of biomolecular condensates in enhancer-promoter interactions and their effects on the dynamics of transcriptional bursting.

## Introduction

Transcriptional bursting has become a popular concept in recent years. It refers to the strongly varying transcriptional output from active genes, where RNA is transcribed in bursts that are relatively short compared with interspersing periods of silence [1].

The dialogue between theory and experimentation has been very fruitful for transcriptional bursting and has cross-fertilized the disciplines. The topic has arguably become a classical case for the application of stochastic modelling in biology [2] and experimental results and novel wet lab techniques continue to inspire mathematical and computational approaches (e.g. [3–5]).

It is intuitively clear that individual transcriptional events must be subject to some degree of variability and are unlikely to be timed precisely like a clockwork. However, the observed dynamics of transcription are significantly more complex than what would be expected in theory, even taking account of basic, thermodynamically unavoidable stochasticity.

## Fundamentals

**Figure 1.**
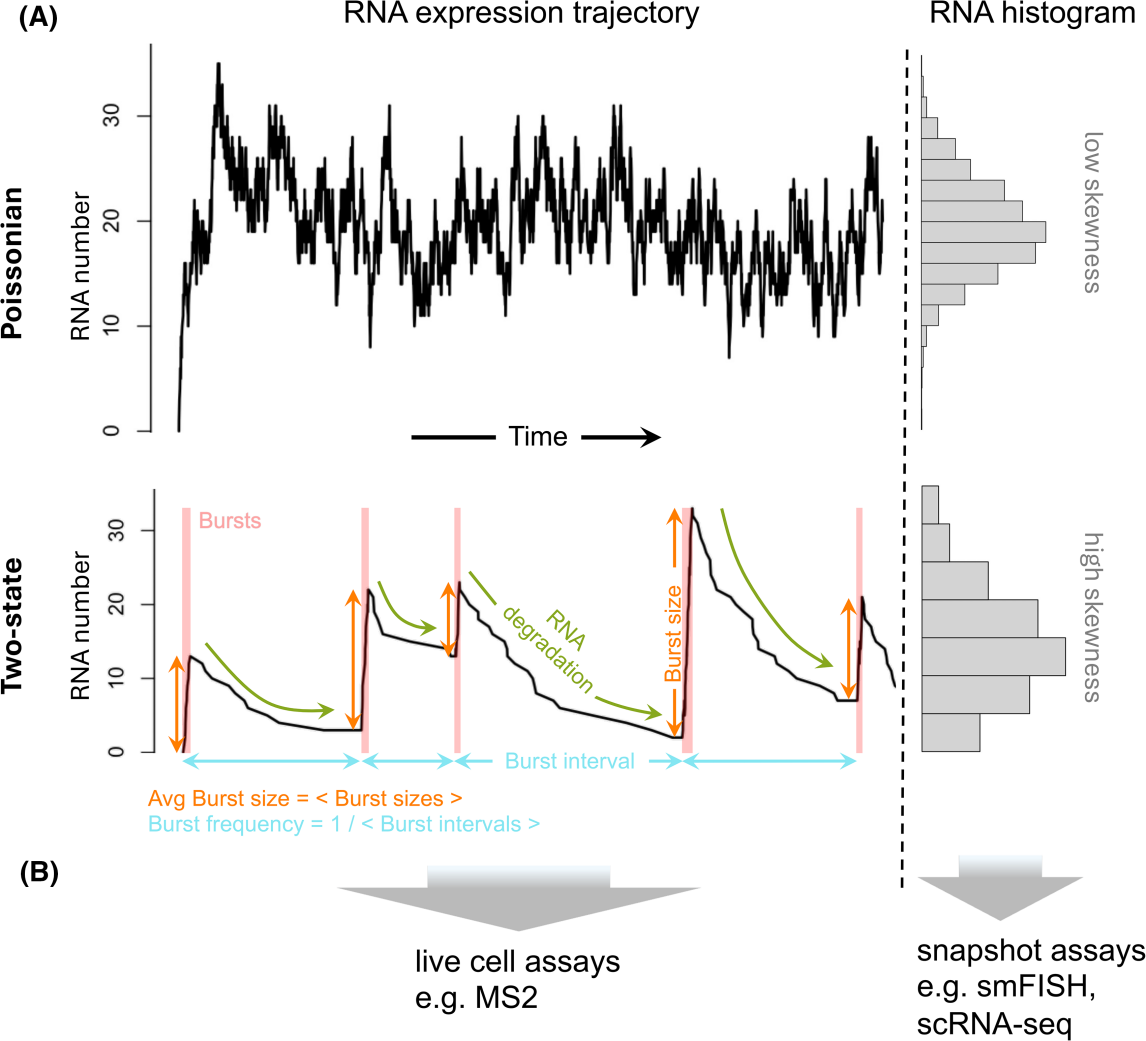
Overview of transcriptional dynamics and experimental techniques to study these different models of transcription. (**A**) Poissonian (top), two-state (bottom). Simulated RNA numbers over time are shown on left, histograms of the same RNA are shown on the right. Note that the two-state model tends to produce more skewed distributions than the Poissonian model. The two-state model is annotated for typical bursting parameters (colours). The Poissonian trajectory and histogram was created with the two-state model by increasing the rate of switching to the ‘on’ state and the degradation rate, making it de facto a one-state model. (**B**) experimental assays usually either track transcription over time in live cells or infer parameters from RNA distributions across individual cells at one timepoint. The arrows relate part A of the figure to the experimental approaches, i.e., trajectory recordings with live cell assays and histogram data collection with snapshot assays, respectively.

Physics allows derivation of the expected waiting times between individual transcriptions from first principles [6]. This is in principle equivalent to the assumption that mass action kinetics drive the biochemical reactions underlying the process. It predicts exponentially distributed waiting times between events, even when considering a great number of different factors interacting. A mathematical consequence of this is that the number of transcription events in a given time interval follows a Poisson distribution (Figure 1A).

A Poisson distribution therefore is the ‘minimal model’ to be expected for the number of RNAs to be transcribed within some period of time and also the number of RNAs to be found in a cell at any time — adding a degradation reaction per above reasoning maintains the Poissonian statistics (Figure 1A).

The Poisson distribution is also ‘minimal’ in the sense that its dispersion scales in a simple way: its variance is always equal to its mean, which also serves as a simple test; any RNA distribution not satisfying this equality cannot have resulted from a Poisson process.

## Prevalence of bursting

Poissonian expression appears rare; experimental evidence suggests that transcriptional bursting is nearly universal. It was found in many different species, including *Escherichia coli* [7], budding yeast [8], *Dictyostelium* [9], *Caenorhabditis elegans* [10], fly [11], mouse [12], humans [13]. In eukaryotes, most of the studies on this topic focus on RNA polymerase II (PolII), but Pol I also exhibits bursting [14].

The ubiquity of burst-like expression is surprising, given how different the transcription machineries in these diverse species are. A possible unifying explanation might be the contribution of DNA structure to bursts; in *E. coli*, DNA supercoiling was shown to build up upon transcription, leading to a block in transcription that can be released by gyrase binding, yielding burst-like dynamics [15]. A theoretical exploration of this in human cells supports this concept [16], and a recent study in budding yeast demonstrates how supercoils inhibit transcription of neighbouring genes. Experimental extension of these findings to human systems and filling in molecular details will require further studies, but this will be challenging due to the difficulty of measuring supercoils in single cells [17].

The wide occurrence of transcriptional bursting and its suspected connection to molecular mechanisms involved in organizing transcription has made it a popular subject of study [18]. Many other theories besides supercoiling have been proposed to explain the underlying (at least partial) causes, some of which are detailed below.

It should be noted, though, that not all genes exhibit burst-like dynamics. An often-cited study from yeast presents examples for Poissonian expression [8]. Another, more recent study suggests that constitutively expressed genes in fission yeast can exhibit even sub-Poissonian statistics, indicating a more complex and varied landscape of low-noise gene expression than previously assumed [19].

There are also some discrepancies when different experimental techniques are considered; transcriptional dynamics are frequently assessed by either live cell imaging that follows transcription from individual loci, or by counting a gene's transcripts in sufficient numbers of individual cells (Figure 1B).

The former is typically an assay such as MS2 [20], which detects single RNAs via reporter proteins that can bind engineered sequences in the transcripts, leading to the local enrichment of fluorescent signal beyond a detection threshold. The latter is often accomplished with an assay such as smFISH, based on hybridization of large numbers of fluorescently labelled DNA probes to target RNAs in fixed cells, again achieving single molecule sensitivity [21].

Both approaches have yielded ample evidence for burst-like transcription. However, a newer experimental development based on the ‘counting’ strategy, single cell RNA-sequencing (scRNA-seq), has produced some conflicting results in regard to the prevalence of bursting; scRNA-seq is theoretically able to output transcriptome-wide expression levels in individual cells. The resulting distributions of RNAs can be used to infer transcriptional dynamics of the genes that expressed them, similar to the way smFISH is employed for this purpose. Prominent scRNA-seq studies report on vast numbers of genes that exhibit Poissonian rather than burst-like expression [22], unlike with smFISH/MS2, where rarely any non-bursting genes are found.

Comparisons of smFISH and scRNA-seq data for the same genes suggest a tacit recognition of inconsistencies that might derive from technical differences in the assays [23]. scRNA-seq is subject to substantial technical noise and requires models to account for lost RNA during sample preparation (‘drop-outs’) [24,25]. This is a potential source of errors and bias that is not easily fixed by boosting sequencing depth due to the losses occurring before this step, but it is not clear if smFISH/MS2 is immune to such problems; it has been argued that the scRNA-seq drop-outs’ origins are biological, not technical [26], while smFISH is known to heavily depend on thresholding associated with spot counting algorithms [27]. A more systematic study of this issue could be helpful to resolve these discrepancies.

## The ‘two states’

Genes that exhibit transcriptional bursting are usually envisioned to be in one of two states; transcription occurs in the ‘on’ state only, while the ‘off’ state is transcriptionally silent. This model, used as both, a ‘cartoon’ like image to simplify conceptions of gene expression, as well as a mathematically precise formalism, had been in use even before burst-like expression achieved recognition; a classical theoretical study from 1995 by Peccoud and Ycart [28] obtained basic results for the two-state model before experimental works firmly established transcriptional bursting as a widespread phenomenon. The Peccoud and Ycart model has remained the standard approach to characterize bursting in the decades since.

The two-state model is an abstraction and simplification of the actual molecular processes at work. It is not as readily obtained from first principles as the minimal model. Rather, the two states are introduced as postulates and biological understanding is drawn upon to give meaning to them. It can be regarded as top-down compared with the Poissonian bottom-up approach.

It should also be noted that the two-state model includes the minimal model as a special case; with certain parameter settings, such as a high rate of switching to ‘on’ compared with the other rates, thus producing a permanent ‘on’ state, a Poisson distribution of RNA results [1] (Figure 1A).

Models with higher numbers of states have been used in several studies to provide better fits to data, as reviewed in [18]. Usually, these extra states account for different promoter conditions and/or ‘refractory’ periods. It is unclear whether this is universal, but might depend on the system studied [18]. The majority of works appears to favour two-state models, with the resolution of experimental assays and parsimony considerations probably playing a role here.

## Measuring bursting parameters

The two-state model depends on parameters that determine the propensities for the states to switch, the ‘on’ state to transcribe, and the RNAs to be degraded. These parameters and certain combinations thereof allow interpretation of the transcriptional dynamics in accessible fashion; commonly used descriptors are the average number of RNAs made during a burst, the ‘burst size’ and the number of bursts per time unit, the ‘burst frequency’, along with standard statistics, such as the mean of the resulting RNA distribution.

These parameters can be measured directly with live-cell assays that resolve the timing of the bursts (Figure 1A,B). For ‘snapshot’ assays such as smFISH or scRNA-seq, some quantities such as the burst size can be inferred based on the shape of the RNA distribution across single cells (Figure 1A,B), but time-dependent ones such as the burst frequency cannot and are found only relative to the RNA degradation rate. Recent developments offer a solution to this, though; a variety of protocols have been developed that combine RNA-sequencing with metabolic labelling. In these approaches, a modified uridine, 4-thiouridine (s^4^U), is added to cells for a specified period of time. The s^4^U gets incorporated into newly transcribed RNA, thus enabling the latter's identification. This is achieved either by purifying (and sequencing) the RNA through targeting the s^4^U tags or by chemically converting the s^4^U's in it, which then tend to appear as cytosines rather than adenines upon sequencing [29]. Relating the quantity of s^4^U + RNA to the s^4^U pulse time allows estimation of the RNA's turnover. Combining this with single cell resolution, i.e. scRNA-seq, allows derivation of the burst frequency in time units and also boosts precision of the burst size's estimate [30].

In many systems, modulations of these bursting parameters under different conditions have been studied in the hope to learn more about the mechanistic backgrounds of the observed transcriptional dynamics.

## Mechanistic insights

Various mechanisms have been proposed to be at the heart of burst-like dynamics of transcription in eukaryotic cells. As mentioned earlier, these potentially include supercoiling, along with transcription factor interactions, assembly kinetics of the core transcriptional machinery, polymerase pausing, or chromatin state changes, and have been reviewed in detail elsewhere [18,31–33].

In many settings, perturbing one or more of these mechanisms modulates bursting by changing burst sizes or frequencies, but, curiously, no study seems to exist that reports a complete reversal to a Poissonian expression mode. Some factors that could plausibly be identified with the two states of the theoretical models, such as certain chromatin states, clearly contribute to transcriptional dynamics, while causal roles as direct drivers of bursting are less obvious [18,31,33]; a systematic study by Suter et al. [34] found inhibition of histone acetylation to modulate bursting in gene-specific ways, while the ‘activating’ H3K4 trimethylation appears to contribute to the inheritance of bursting patterns to daughter cells more generally [35], for example. Other studies report on chromatin accessibility affecting burst frequency [36]. For a detailed review of epigenetics and bursting, see [37].

In other cases, timescales are mismatched; in multicellular organisms, the ‘on’ times usually last a few minutes, with the ‘off’ times roughly an order of magnitude longer, while residence times of transcriptional factors tend to be less than one minute [32]. No ‘smoking gun’ type of evidence has materialized yet for the origins of eukaryotic transcriptional bursts.

A wave of new studies into the causality of bursting recently emerged at the nexus of chromatin topology and liquid-liquid phase separation (LLPS). The latter refers to a type of factor aggregation driven by forces with mesoscopic character, such as mixing-energy and -entropy trade-offs and/or polymer physics, rather than site-specific high-affinity interactions. It has emerged as an important biological mechanism [38] although its true significance in transcription is subject to debate [39].

Interactions between enhancers and their target genes are believed to require looping of chromatin, given the occasionally very large distances when their locations on linear DNA are considered [40]. Loop extrusion, mediated by the cohesin complex, is one of the key players in enhancer-promoter communication. It likely functions by scanning a confined region within a TAD (Topologically Associating Domains), delineated by CTCF boundaries [41]. This brings enhancers close to cognate promoters, facilitating targeted transcription and ensuring enhancer specificity. However, enhancer-promoter interaction complexity extends beyond TADs, involving factors such as gene loops and chromatin accessibility; while TADs themselves could be regarded as epigenetic features [42], CTCF affects the local histone modifications [43] and enhancers exhibit characteristic histone modification profiles [44]. The precise mechanism through which distal DNA elements interact within the nuclear space remains an active area of research. How exactly this is accomplished remains unclear, but in many cases, biomolecular condensates — formed as result of LLPS or other means [39] — have been found at enhancers [45–48], and such condensates are able to restructure chromatin [49,50].

The transient nature of enhancer — target gene contacts [32] suggests their involvement in the interrupted nature of transcription, and studies have indeed explored this and found bursting parameter modulation in many cases [51–57]. At the same time, the repeated transcription in rapid succession that takes place during a burst might conceivably require a microenvironment that facilitates this, including high concentrations of and/or clusters of transcriptional factors. The presence of condensates at enhancers and/or transcribing genes appears to satisfy these criteria in principle.

**Figure 2.**
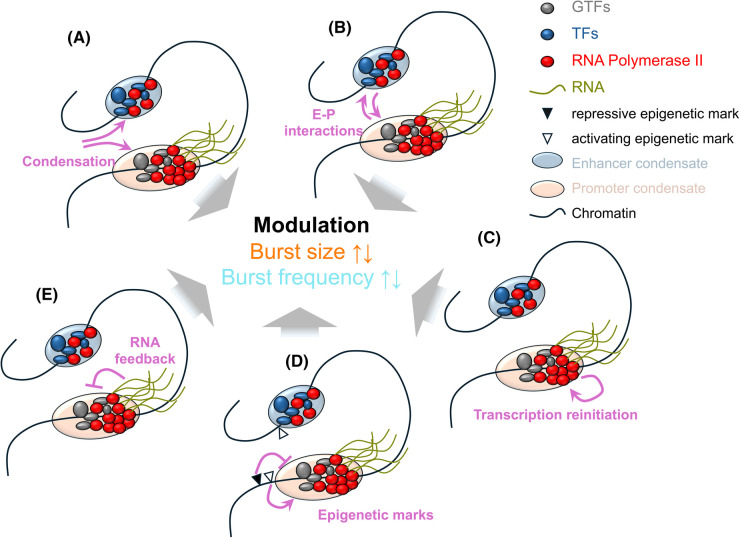
Schematic illustration of potential mechanisms contributing individually or jointly to the modulation of bursting parameters. Included are formation of biomolecular condensates themselves (**A**), transient enhancer-promoter (E-P) interactions leading to infrequent transcriptional activation and thus bursts (**B**), transcription reinitiation and thus repeated transcription contributing to bursts (**C**), changing epigenetic marks contributing to interrupted transcriptional activity (**D**), and RNA feedback leading to assembly and disassembly of transcription-facilitating condensates and thus interrupted transcription (**E**). Selected example references for these are [36,62–64] and [60], respectively. The circles symbolize enhancer and promoter condensates, shaded in light blue and light brown, respectively, which are not necessarily separate or of different size, but sometimes are [62]. GTFs, General Transcription Factors, TFs, Transcription factors, and RNA polymerase II (PolII) are shown in grey, blue, and red, respectively. Black and green lines are chromatin and RNA, respectively. Epigenetic marks are shown as triangles as indicated. The illustrations are showing active genes that have been transcribing in burst-like on/off fashion, with recent transcripts (green) still close to the gene. Enhancers are dominated by TFs and feature low numbers of PolII that potentially transcribe enhancer RNA at low rates, while promoters are dominated by GTFs and large numbers of PolII.

A number of recent works unifies some of these concepts [58], reminiscent of earlier conceptualizations of transcription [59]. Henninger et al. propose that formation of transcriptional condensates at promoters and enhancers is subject to feedback with transcribed RNA; high local production of RNA dissolves condensates, leading to stops in transcription and a need to reset the system at lower RNA levels, inducing burst-like dynamics [60]. The same RNA-based feedback might affect promoter-enhancer interactions, with similar effects on transcriptional dynamics [61]. Enhancer- and promoter-based condensates were also shown to directly modulate a gene's bursting behaviour when in proximity [62,63]. Our own work suggests repeated reinitiation of transcription to contribute to bursting [64], potentially involving condensate formation to trap polymerases in a local genic niche; clustering of polymerases at transcribing genes is now firmly established [65–67]. Figure 2 shows a schematic summary of some of these effects.

While the role of biomolecular condensates and/or LLPS in the organization of transcription is an active area of research, in some cases effects on transcriptional dynamics are clear, whereas other findings appear locus-specific and do not generalize, or the absence of such effects is apparent. A prominent example is Trojanowski et al. [68], who demonstrate that the propensity of transcription factors to undergo LLPS correlates with their activation strength, but their actual condensation itself does not. Similarly, a study employing rapid depletion of CTCF demonstrates that the latter dissolves condensates consisting of PolII and other transcriptional factors, but leaves global transcriptional activity largely intact [69]. Stortz et al. [70] provide a good summary of the current understanding.

## Perspectives

- Transcriptional bursting is nearly universal and its underlying mechanisms are still unclear.
- Some experimental techniques produce conflicting results and no perturbation of bursting has produced a reversal to a Poissonian expression mode.
- Epigenetic chromatin marks influence transcriptional bursting directly or indirectly.
- The novel integration of chromatin topology and condensate biology might be key to advancing the field.

## Open Access

Open access for this article was enabled by the participation of University of Warwick in an all-inclusive *Read & Publish* agreement with Portland Press and the Biochemical Society under a transformative agreement with JISC.

## Abbreviations

LLPS: liquid-liquid phase separation
PolII: polymerase II
TAD: Topologically Associating Domain
